# Horizontal Cells, the Odd Ones Out in the Retina, Give Insights into Development and Disease

**DOI:** 10.3389/fnana.2016.00077

**Published:** 2016-07-19

**Authors:** Henrik Boije, Shahrzad Shirazi Fard, Per-Henrik Edqvist, Finn Hallböök

**Affiliations:** ^1^Department of Neuroscience, Uppsala UniversityUppsala, Sweden; ^2^Department of Immunology, Genetics and Pathology, Uppsala UniversityUppsala, Sweden

**Keywords:** cell fate, cell cycle regulation, chicken, development, DNA damage, neuronal subtype, retinoblastoma, zebrafish

## Abstract

Thorough investigation of a neuronal population can help reveal key aspects regarding the nervous system and its development. The retinal horizontal cells have several extraordinary features making them particularly interesting for addressing questions regarding fate assignment and subtype specification. In this review we discuss and summarize data concerning the formation and diversity of horizontal cells, how morphology is correlated to molecular markers, and how fate assignment separates the horizontal lineage from the lineages of other retinal cell types. We discuss the novel and unique features of the final cell cycle of horizontal cell progenitors and how they may relate to retinoblastoma carcinogenesis.

## A brief introduction to horizontal cells

Multipotent retinal progenitor cells (RPCs) in the vertebrate optic cup give rise to the five types of neurons and one type of glia that make up the mature retina (reviewed in Masland, 2001a; Marquardt and Gruss, 2002; Boije et al., 2014). The neuronal cell types: photoreceptors, horizontal, bipolar, amacrine, and ganglion cells, can be further divided into subtypes based on various morphological, functional, and molecular criteria (Masland, 2001b). During the Nineteenth century the Spanish neuroscientist Santiago Ramón y Cajal was the first to classify the retinal neurons and some of their subtypes based on morphological criteria (Ramón y Cajal, 1972). Since then, molecular and functional definitions have often been found to correlate with the morphological classifications thereby validating the oldest taxonomy.

Horizontal cells (HCs) have been identified in all vertebrate retinas from fish to man (Gallego, 1971, 1982, 1986; Peichl et al., 1998). They were found to be the source of the puzzling S-potentials, where hyperpolarization occurs as a response to light stimulus (Svaetichin, 1953; Werblin and Dowling, 1969; Kaneko, 1970). HCs facilitate both long and short range interactions between photoreceptors (PRs) and through inhibitory feed-back they aid in contrast enhancement and color opponency (Twig et al., 2003). There are several reasons why HCs are of particular interest. They have clearly distinguishable subtypes, both by morphological criteria and by molecular markers, allowing the study of subtype formation. Unlike many other cell types in the retina, the final number of HCs is not adjusted by apoptosis (Mayordomo, 2001; Edqvist et al., 2008). HCs undergo a unique bi-directional migration, resulting in a stopover at the basal side of the retina before arriving at their predestined position close to the PRs (Edqvist and Hallböök, 2004). Furthermore, in zebrafish and chicken, committed HCs has been shown to undergo non-apical mitoses in a semi-differentiated state (Godinho et al., 2007; Boije et al., 2009). Recent studies in chicken revealed a novel cell cycle behavior by the HCs that includes endoreplication leading to heteroploidy (Shirazi Fard et al., 2013). Moreover, HCs can divide in the presence of DNA damage and are able to develop into retinoblastoma in a murine model (Ajioka et al., 2007; Shirazi Fard et al., 2014b). In this review we will discuss the progress made regarding these HC features and how these findings relate to general concepts of development, evolution, and tumor formation.

## Morphological and functional diversity of horizontal cells

**Figure 1:**
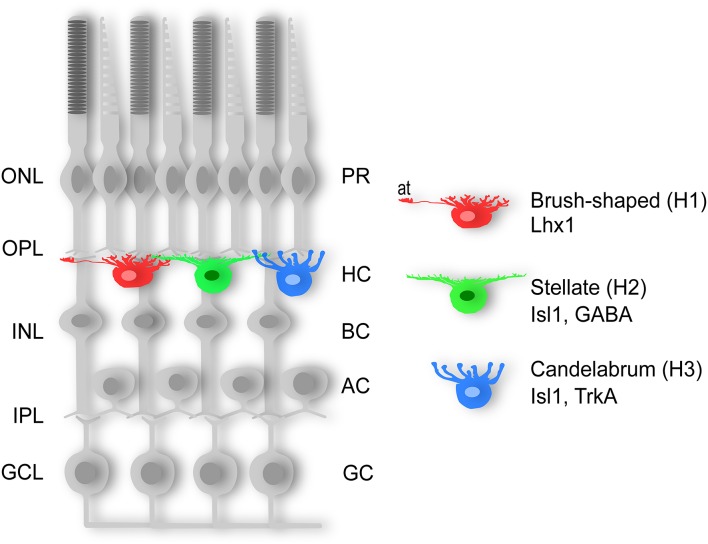
**Three horizontal cell types**. Schematic diagram of the three subtypes of chicken horizontal cells. The “brush-shaped” (H1) is axon-bearing and expresses Lhx1 (Lim1), whereas the “stellate” (H2) and the “candelabrum-shaped” (H3) are both axon-less and express Isl1. AC; amacrine cell, BC; bipolar cell, GC; ganglion cell, GCL; ganglion cell layer, H1-3; horizontal cell type 1-3, HC; horizontal cell, INL; inner nuclear layer, IPL; inner plexiform layer, ONL; outer nuclear layer, OPL; outer plexiform layer, PR; photoreceptor.

Horizontal cells can be divided into axon-bearing and axon-less subtypes, a feature that appears to be conserved in the vertebrate lineage with few known exceptions (Gallego, 1986; Peichl et al., 1998; Table 1). The emerging pattern for all vertebrate retinas examined so far is that the axon-bearing HC is universal, and that any additional HC subtype(s) adhere to the axon-less population. In the chicken retina, three different HC subtypes have been described based on morphology; the “brush-shaped” (H1) is axon-bearing, whereas the “stellate” (H2) and the “candelabrum-shaped” (H3) are both axon-less (Figure 1; Genis-Galves et al., 1979; Gallego, 1986). In some species these HC subtypes are referred to as A-, B-, and C-type (Table 1). As will be discussed below, this morphological classification has since been found to correlate with the expression of unique molecular markers.

**Table 1:** **Species comparison of horizontal cell subtypes**.

**Species**	**H1 B-type Axon-bearing**	**H2 A-type Axon-less**	**H3–H4 C-type Additional subtypes**	**Comment**	**References**
**FISH**					
Zebrafish	x	x	xx	Include Isl1+ HCs	Connaughton et al., 2004; Song et al., 2008; Zhang et al., 2013
White perch	x	x	xx		Dowling et al., 1985
Carp	x	x	xx	H1-2 are Cx35/36+, not H4	Liu et al., 2009
Shark(s)	x	x	x	Calretinin and GABA double and single labeling subtypes	Ferreiro-Galve et al., 2010; Schieber et al., 2012
Stingray	x	x	x		Toyoda et al., 1978
Sea lamprey	x	x			Villar-Cheda et al., 2006
Deep sea eel	x			Rod-only retina	Hirt and Wagner, 2005
Goldfish	xxx			Three subtypes, all have axons	Kamiji et al., 2012
**REPTILES**					
Turtle(s)	x	x	xx	Isl1, GABA, Calretinin, Calbindin in subpopulations	Leeper, 1978; Francisco-Morcillo et al., 2006
Frog(s)	x	x	x	Include Isl1+ HCs	Ogden et al., 1984, 1985; Álvarez-Hernán et al., 2013
Mudpuppy	x	x	x		Kim and Miller, 1992
Tiger salamander	x	x		A-type: Calretinin+ B-type: GABA+	Zhang J. et al., 2006
Chameleon	?	?		One Gad65+ subpopulation	Bennis et al., 2003
**BIRDS**					
Pigeon	x	x	xx		Mariani, 1987
Chicken	x	x	x	Include Lhx1, Isl1, GABA and TrkA cells	Gallego et al., 1975; Edqvist et al., 2008
Owl	x	x			Tarrés et al., 1986
**MARSUPIALS**					
Didelphis opossum	x	x	x	Diurnal	Hokoc et al., 1993
Brush-tailed possum	x	x			Harman, 1994
Wallaby	x	x			Harman and Ferguson, 1994
Brazillian opossum	x			Nocturnal	Lyser et al., 1999
**VARIOUS MAMMALS**					
Horse, Ass, Zebra	x	x			Sandmann et al., 1996a
Sheep, Ox	x	x			Sandmann et al., 1996b
Cat	x	x			Boycott et al., 1978; Vardi et al., 1994
Pig	x	x		Include Isl1+ HCs	Sandmann et al., 1996b; Guduric-Fuchs et al., 2009
Rabbit	x	x	?	A-type: Cx50+ and NF+ B-type: Cx57+ and Calbindin+ Third type: “Elongated A-type”?	Bloomfield and Miller, 1982; Dacheux and Raviola, 1982; Silveira et al., 1989; Famiglietti, 1990; Lyser et al., 1994; Hack and Peichl, 1999; Pan et al., 2012
Tree shrew	x	x		A-type is GFAP+	Müller and Peichl, 1993; Knabe and Kuhn, 2000
**RODENTS**					
Squirrel(s)	x	x			West, 1978; Leeper and Charlton, 1985; Linberg et al., 1996; Cuenca et al., 2002
Guinea pig	x	x			Peichl and González-Soriano, 1994; Loeliger and Rees, 2005
Agouti	x	x		Diurnal rodent	Silveira et al., 1989; de Lima et al., 2005
Capybara	?	x			Silveira et al., 1989
Naked mole rat	x	?		1–2 HCs detected in a regressive eye	Mills and Catania, 2004
Rat	x				Peichl and González-Soriano, 1994
Mouse	x			Lhx1 positive, Isl1 negative	Peichl and González-Soriano, 1994; Liu et al., 2000; Hombach et al., 2004; Elshatory et al., 2007
**PRIMATES**					
Cebus monkey	x	x	x		dos Reis et al., 2002; Dos Santos et al., 2005
Owl monkey	x	x	x	Nocturnal	Dos Santos et al., 2005
Macaca monkey	x	x			Wässle et al., 2000; Hendrickson et al., 2007
Marmoset monkey	x	x			Chan et al., 1997
Human	xxx			Three types, all have axons	Kolb et al., 1992; Kolb, 1995; Nag and Wadhwa, 2001

“*x” denotes one HC subtype being present in the species*.

The division into axon-bearing and axon-less HC subtypes has also been suggested to reflect a functional difference where the axon-terminus of H1 HCs mainly connects to rod PRs, whereas the dendritic tree of all subtypes exclusively forms connections with cone PRs (Zhang A. -J. et al., 2006; Zhang J. et al., 2006). Although there is no clear-cut relationship between a low cone/rod ratio and the number of HC subtypes (reviewed in Peichl et al., 1998), the difference in the number of HC subtypes among different species seem to be loosely correlated with the relative numbers of cone and rod PRs. In cone-rich retinas (e.g., the chicken retina), two types of axon-less HCs are present alongside the axon-bearing HCs (Genis-Galves et al., 1979; Gallego, 1986). In contrast, rod-dominated retinas, such as most mammalian retinas (Ahnelt and Kolb, 2000), have only one type of axon-less HC. Extremely rod-dominated retinas, such as the mouse and rat retina, with merely 1–3% cones (Szél et al., 1996; Peichl, 2005), lack the axon-less HC subtypes altogether (Peichl and González-Soriano, 1994). Examples of species with only the axon-bearing HC subtype have been described in such diverse groups as fish, marsupials, and mammals (Peichl and González-Soriano, 1994; Lyser et al., 1999; Hirt and Wagner, 2005; Table 1). Thus, it may be that the loss of axon-less HCs in highly rod-dominated retinas is a result of adaptations to a nocturnal lifestyle, since few cones would make the “cone-specialized” axon-less HCs obsolete.

## Molecular markers for horizontal cell subtypes

Data from our lab and others have shown that all HCs in the chicken retina express the homeodomain transcription factors (TF) Prox1 and Pax6 whereas the LIM/homeodomain TFs Lhx1 (Lim1) and Isl1 are both expressed in half the HC population in a non-overlapping manner (Edqvist et al., 2006, 2008; Fischer et al., 2007). Specifically, the axon-bearing HCs express Lhx1, fibroblast growth factor 19 (Francisco-Morcillo et al., 2005; Okamoto et al., 2009), and calretinin (Edqvist et al., 2008) while both axon-less subtypes lack the expression of these markers, but instead expresses Isl1 (Edqvist et al., 2008; Figure 1). Based on these observations, Lhx1 and Isl1 have emerged as markers for the axon-bearing and axon-less HC subpopulations, respectively.

The axon-less H2 and H3 populations can be distinguished based on their expression of GABA and the nerve growth factor receptor tyrosine kinase TrkA, respectively (Karlsson et al., 1998, 2001; Edqvist and Hallböök, 2004; Boije et al., 2008). Although HCs are often referred to as GABAergic inhibitory interneurons GABA is only present in the H1 and H2 subtypes (Edqvist and Hallböök, 2004; Boije et al., 2008). Certain cadherins and connexins are also expressed in HCs in a subtype-specific pattern (Tanabe et al., 2004, 2006; Puller and Haverkamp, 2011; Pan et al., 2012).

Although Lhx1 was identified as a HC-specific marker in the retina (Liu et al., 2000), the sub-type specific quality of Lhx1 was initially unknown. Isl1 on the other hand is not uniquely expressed in HCs but is also expressed in ganglion cells, certain amacrine cells (ACs), and bipolar cells. However, the combination of Prox1 and Isl1 is exclusive to HCs. When Isl1 was first described as a novel HC subtype-marker in chicken (Edqvist et al., 2006), it was uncertain whether the Lhx1/Isl1 division of chicken HCs into axon-bearing and axon-less subtypes was an evolutionarily conserved feature in other species. Subsequent studies show that Isl1 is expressed in HCs in retinas of various species including fish, frogs, turtles, and pigs (Francisco-Morcillo et al., 2006; Guduric-Fuchs et al., 2009; Álvarez-Hernán et al., 2013; Zhang et al., 2013; Table 1). Taken together, this suggests an evolutionary conserved division of Lhx1 and Isl1 into different HC subtypes that confirms the morphologically based subdivision.

## Fate specification of horizontal cells rely on FoxN4 and Ptf1a

Although several bHLH and homeodomain TFs (e.g., Ascl3, Ngn2, NeuroD1, Atoh3, Pax6, Onecut1 and 2, and Six3) have been associated with HC genesis, all of these required combinatorial over-expression or knock-out in order to affect the HC population (Inoue et al., 2002; Akagi et al., 2004; Wang and Harris, 2005; Wu et al., 2013). Prox1 has been proposed to be crucial for the generation of HCs, though its relatively late onset of expression suggests that it is more likely involved during maturation rather than fate commitment (Dyer et al., 2003; Boije et al., 2008, 2009). However, the complete abolishment of HCs in retinas lacking the winged helix TF FoxN4 indicated that there indeed may be a key fate determinant for HCs (Li et al., 2004). The expression pattern of FoxN4 in the developing retina is conserved in mouse, frog, chicken, and zebrafish (Gouge et al., 2001; Danilova et al., 2004; Schuff et al., 2006; Boije et al., 2008). FoxN4 is expressed throughout the neural epithelium of the optic cup but is upregulated in a subset of cells that are Lhx1 positive suggesting a commitment down the HC path. Knock-out of the bHLH Ptf1a, a down-stream target of FoxN4, turned out to mimic the phenotype of the FoxN4 mutant with a complete loss of HCs and a drastic decrease of ACs (Li et al., 2004; Fujitani et al., 2006). Subsequent studies of FoxN4 and Ptf1a revealed that they are not only required, but also sufficient, for the generation of HCs and ACs (Dullin et al., 2007; Lelièvre et al., 2011; Boije et al., 2013).

Clearly, both FoxN4 and Ptf1a are required for the formation of HCs, but equally so for the generation of ACs. How does this FoxN4/Ptf1a-lineage divide into two closely related, but functionally distinct, cell types? In mice, which only possess the axon-bearing HC subtype, Lhx1 appeared as an obvious candidate for lineage diversification since it uniquely labels all HCs. Conditional Lhx1 mutant mice showed that its ablation caused ectopic HCs with a morphology resembling that of ACs (Poché et al., 2007). Expression of Ptf1a and Prox1 was initiated as in wild type HCs but the retrograde migration from the basal side (see paragraph regarding HC migration) was not performed. Although the ectopic HCs adopted an AC morphology they did not express AC specific markers suggesting proper fate commitment had occurred but that they had an erroneous laminar position (Poché et al., 2007). This indicated that Lhx1 may be a crucial homeodomain protein for the positioning of HCs in the mouse retina. It also indicated that Lhx1 expression is not required for the expression of either Ptf1a or Prox1. The fact that Lhx1 expression is observed during apical mitoses in the chicken retina, prior to the onset of Ptf1a expression, highlights a common mis-perception of the temporal aspect of these genes (Boije et al., 2013). Instead this suggests a process where Ptf1a and Lhx1 are both required but independently regulated in order to specify HCs.

**Figure 2:**
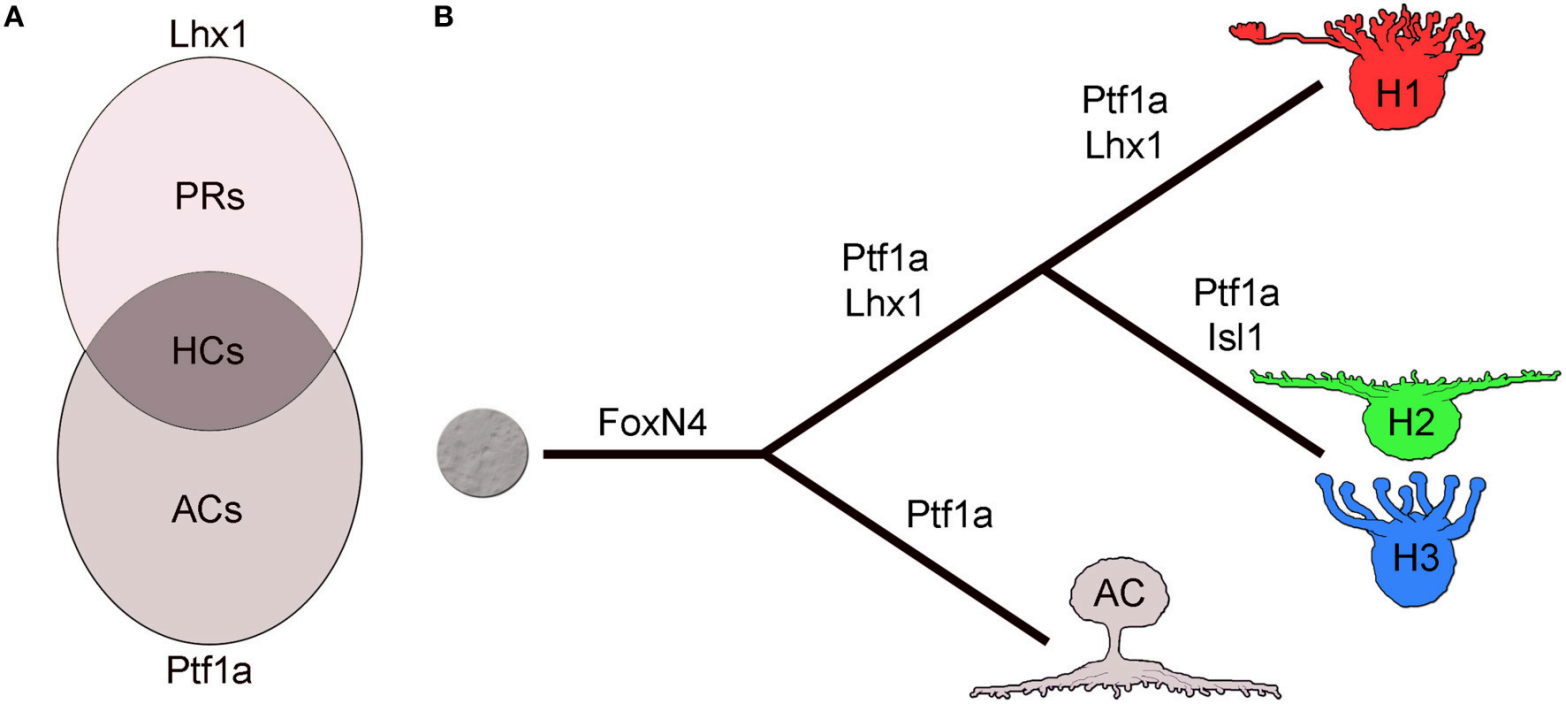
**Horizontal cell fate and cell type formation. (A)** Horizontal cell fate assignment can be regarded as the intersectional population of the independent expression of Ptf1a and Lhx1. **(B)** The pathway to horizontal cells is linked to amacrine cells by the expression of FoxN4 and Ptf1a while Lhx1 separates the HC lineage from ACs. Isl1 blocks Lhx1 expression to form the axon-less subtypes. AC; amacrine cell, HC; horizontal cell, H1-3; horizontal cell type 1-3, PR; photoreceptor.

Lineage analysis in the zebrafish retina using transgenic lines driving fluorescent reporter genes under the promoters of Lhx1 and Ptf1a suggests how independent expression of key factors may regulate HC fate assignment. Two things stand out regarding the Lhx1-lineage: firstly, all HC subtypes arise from the Lhx1-lineage, and secondly, most cells of this lineage do not become HCs but adopt the PR fate (Boije et al., 2015). A recent study by the Wong lab further stresses this close relationship between HCs and PRs (Suzuki et al., 2013). Quantification of the different lineages suggested that the generation of the HCs could be explained as the intersectional population of the independent expression of Lhx1 and Ptf1a (Figure 2A; Boije et al., 2015), while cells that expressed only Ptf1a or only Lhx1 become ACs or PRs, respectively. Recalling the conditional Lhx1 mutant mice, there seem to be two pieces to the puzzle; Ptf1a to drive differentiation and Lhx1 to govern positioning.

## Horizontal cell subtype formation

As the presence of both axon-bearing and axon-less HCs seems to be an evolutionarily conserved feature, it is natural to question how these subtypes arise. While FoxN4 specifies subtype identity in the spinal cord this does not appear to be the case in the retina (Li et al., 2005). Knock-out or over-expression of FoxN4, affected the fate assignment of V2a vs. V2b spinal interneurons via the Notch-Delta pathway (Li et al., 2005; Del Barrio et al., 2007). In the retina however, FoxN4 activates Dll4-Notch signaling suppressing PR fate but it does not seem to play a role in HC subtype formation as both Lhx1 positive (+) and Isl1+ HCs were lost in the mutant and both subtypes were generated by FoxN4 overexpression (Boije et al., 2013). Similarly, Ptf1a has been suggested to determine GABAergic cell fate in the spinal cord and in the cerebellum (Glasgow et al., 2005; Hoshino et al., 2005). In contrast to the mouse retina, which only has the Lhx1 expressing GABAergic HC subtype, the chicken retina also has non GABAergic HCs expressing Isl1. However, Ptf1a overexpression led to an increase in both GABAergic Lhx1 positive HCs and in TrkA, Isl1 double positive HC subtypes (Lelièvre et al., 2011). This suggests that Ptf1a is involved in assigning inhibitory neurons in the retina, rather than specifying GABAergic subtypes.

Since recent lineage analysis implies a common origin of the HC subtypes in zebrafish there may be intrinsic and/or extrinsic factors responsible for the progression from generating one subtype to generating the next. Birth-dating analysis in the chicken retina has revealed that the axon-bearing population is born roughly 1 day prior to the axon-less subtypes (Edqvist et al., 2008). Furthermore, studies in the chicken retina have shown that Isl1, which is the molecular signature of the later born axon-less HC subtypes, is necessary and sufficient to down-regulate the expression of Lhx1 and inducing the phenotypic trait of axon-less HCs (Suga et al., 2009). Overexpression of Isl1 in HCs residing in the HC layer represses endogenous Lhx1 expression and cause a subtype fate switch from the H1 morphology to the H2 but not to H3 (Suga et al., 2009). Expression of a dominant negative Lhx1 variant does not cause subtype fate-switch and overexpression of Lhx1 did not affect the proportion of the HC subtypes. During normal development there are occasionally cells that are double labeled for Lhx1 and Isl1 during the migration to the basal side (Boije et al., 2009). Combined, the lineage data and the dominance of Isl1 over Lhx1, suggests that there is a pool of committed HCs that initiates expression of Isl1 causing a subtype fate-switch (Figure 2B). How this is regulated to produce the 50/50 split in the chicken retina remains unknown. Interestingly, in the mouse retina, which only hosts the Lhx1+ subtype, the number of HCs was found to be inversely dependent on the expression of Isl1 (Whitney et al., 2011). This was shown by the identification of an expression quantitative trait locus in the Isl1-gene from genetic analysis of mouse strains that have inherently different HC numbers. These findings are consistent with a role for Isl1 in regulating the formation or population size of the Lhx1+ subtype and such a role has also been found during the formation of other neuronal subtype-populations (Sun et al., 2008).

## Horizontal cell migration

As RPCs pass through the cell cycle their nuclei undergo translocation across the neuroepithelium, a process known as interkinetic nuclear migration, while remaining attached to the apical and basal lamina (Baye and Link, 2007). Mitoses typically occur on the apical side of the neuroepithelium and after a neurogenic division the post-mitotic daughter cells detach and migrate toward their final laminar location (Götz and Huttner, 2005). Based on this, one would expect the newborn HCs to simply migrate the short distance from the apical side, to the outer part of the prospective inner nuclear layer, where they reside in the mature retina. However, HCs deviate from this path by migrating from their site of birth across the width of the neuroepithelium and halt near the prospective ganglion cell layer before migrating back again toward their final laminar location (Edqvist and Hallböök, 2004). This phenomenon has also been described in mouse (Liu et al., 2000) and zebrafish (Chow et al., 2015), and the presence of displaced HCs in various species (cat, chicken, macaque, augoti, rabbit, capybara) suggest that this migration pattern may be an evolutionarily conserved HC feature (Prada et al., 1984; Silveira et al., 1989; Wässle et al., 2000). Subsequent studies in the chicken retina showed that H1, H2, and H3 HCs all undertake the same route of migration, but the migration of H1 and H2/H3 is temporally separated by approximately 1 day (Edqvist et al., 2008). The separation in migration correlates with a similar difference regarding birth-dates, indicating that the subtypes are not generated during the maturation process but rather that cells are born into a specific subtype fate. This notion is supported by the fact that Isl1, which specifies HC-subtype, is already expressed during the birth of HCs. A similar phenomenon is known from AC subtype formation in the rat retina where GABAergic ACs are born 2–3 days before acetylcholine and dopamine expressing ACs (Lee et al., 1999).

Why do HCs in the Lhx1 loss-of-function mouse retina adopt AC morphology in the absence of the retrograde migration despite not adopting AC molecular characteristics (Poché et al., 2007)? This may either reflect that the morphological maturation of HCs solely depends on Lhx1 or that there are environmental cues governing this step. Interestingly, recent results show that wild type HCs transplanted into retinas lacking HCs and ACs also fail the retrograde migration (Boije et al., 2015). These HCs are intrinsically wild type with the competence to migrate back but they never the less fail. This phenomenon may be due to the absence of a non-cell autonomous factor, possibly arising from ACs. Interestingly, there seems to exist some regulatory mechanism in which a certain number of ACs has to be present for the retrograde migration of HCs to occur (Boije et al., 2015). Furthermore, these HCs also adopted an AC morphology and extended processes into the inner plexiform layer. This suggests that HC morphological maturation relies, at least partly, on extrinsic cues. One can hypothesize that there may have been an ancient inhibitory neuron specified by Ptf1a expression and that the addition of Lhx1 has split this population in two, the HCs and the ACs. The migration of HCs may therefore be an evolutionary remnant from when these cells were destined to reside together.

## Heterogeneity during the final cell cycle of horizontal cells

**Figure 3:**
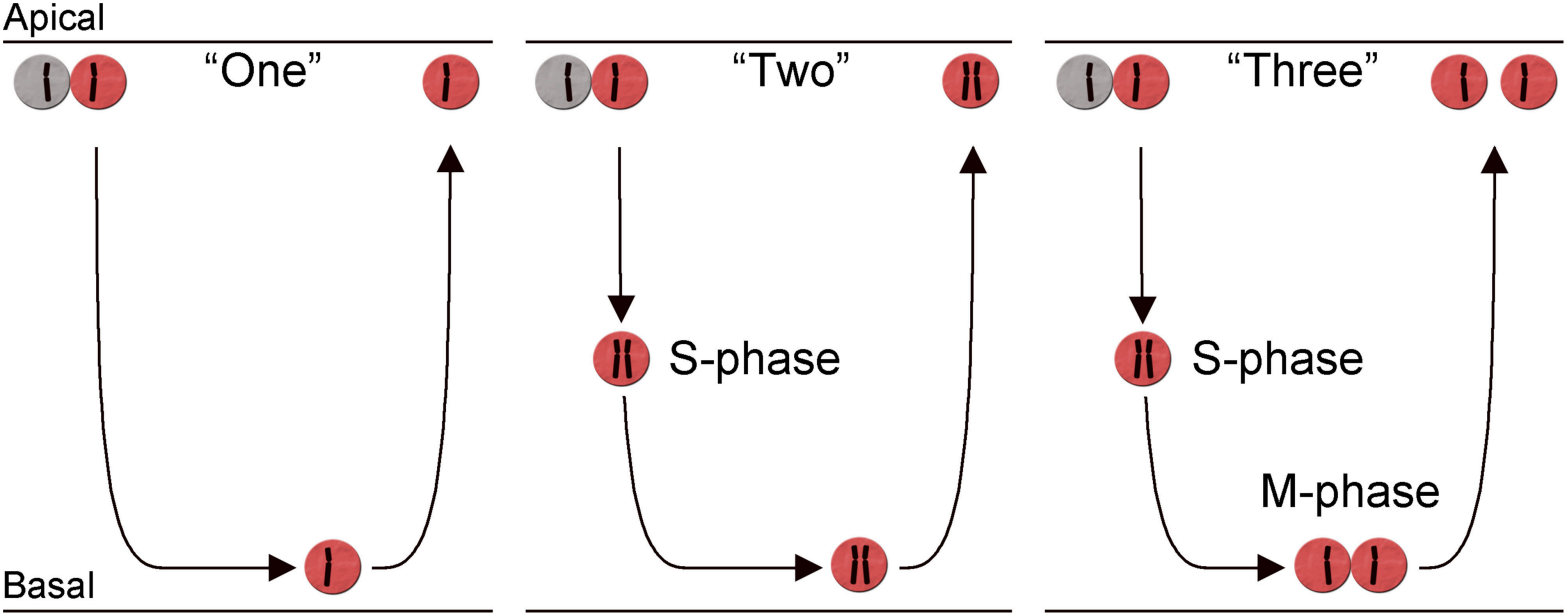
**Heterogeneity in the terminal cell division of horizontal cells**. Schematic diagram showing three different behaviors during the neurogenic cell division of chicken horizontal H1 (Lhx1^+^) cells. The diagram illustrates the retina with mitosis (M-phase) and S-phase indicated in relation to the migration across the retina. The migration is indicated by the arrows and the replicated state of each cell, in the three behaviors is indicated by a schematic chromosome. Two chromosomes indicate a cell with replicated DNA. The three different behaviors are denoted: behavior “One,” “Two,” and “Three” in the main text.

Once a cell undergoes the neurogenic division, the daughter cells become post-mitotic, migrate out to their final laminar position, and initiate differentiation. While this is true for several of the retinal neuronal cell types, chicken HCs can divide once more after having initiated their final migration (Boije et al., 2009). These non-apical mitoses occur on the basal side of the retinal neuroepithelium in a semi differentiated state. However, not all HCs act in this way and three different behaviors have been described during the terminal cell cycle. The first behavior, denoted behavior “one,” resembles that of the RPCs, with an apical mitosis followed by migration and accumulation on the basal side of the retina where they rest for several days before migrating back to the prospective HC layer. HCs with behavior “two” initiate a non-apical neurogenic cell cycle by entering S-phase, however they do not proceed into mitosis and remain with a replicated genome. The presence of such cells was shown by the existence of increased DNA content and double number of chromosomes in post-mitotic HCs (Shirazi Fard et al., 2013). However, the majority of HCs perform a non-apical (basal) mitosis that generates two HCs. Clonal analysis has shown that individually labeled HC progenitors often generated two HCs of the same subtype indicating that the assignment of subtype in many cases occurs prior to the terminal division (Rompani and Cepko, 2008). The non-apical mitoses are denoted behavior “three” and occur later than the behavior “one” and “two”-cells, although there is extensive overlap between the three behaviors (Figure 3). Independent of behavior during the final cell cycle, all HCs undergo retrograde migration and form the prospective HC layer in proximity to the PRs (Shirazi Fard et al., 2013). These behaviors have so far only been carefully studied in the chicken H1 subtype but it is likely that the H2 and H3 HC subtypes have similar behaviors during their neurogenic cell division since they all exhibit basal mitoses in a similar fashion as the H1 HCs do (Boije et al., 2009).

A similar heterogeneity during the neurogenic HC division is seen in the zebrafish retina. HCs divide non-apically on the basal side of the zebrafish retina (Weber et al., 2014) or in the HC layer (Godinho et al., 2007). Such mitoses resemble the behavior “three” cells in the chicken retina. In zebrafish HCs are occasionally generated together with a PR as its sister cell and this pattern may represent the behavior “one” with an apical cell division (He et al., 2012). There have been no reports indicating any heterogeneity during the neurogenic cell divisions of either mouse or rat HCs. They migrate and accumulate on the basal side of the retina and stay on the basal side as post-mitotic cells before their retrograde migration to the prospective HC layer. This conclusion finds support in that the rodent retina only has H1 HCs (Peichl and González-Soriano, 1994; Poché and Reese, 2009) and their behavior during the neurogenic cell division corresponds to behavior “one” cells in the chicken retina (Liu et al., 2000).

Cells that fail to enter mitosis after genome replication, similar to that seen for the behavior “two” HCs, are often associated with DNA damage and an active DNA damage response (DDR; Zhou and Elledge, 2000). However, the arrested chicken HCs are not a result of an active DDR since they do not display phosphorylation of histone H2AX nor Rad51 foci, which are hallmarks of an active DDR and repair (Shirazi Fard et al., 2013). Rather, the heteroploid cells are produced by a mechanism referred to as endoreplication (Edgar and Orr-Weaver, 2001). Overall, endoreplication and its regulation is poorly understood, but it has been linked to cells that have initiated differentiation while remaining in the cell cycle (Zanet et al., 2010). It has also been suggested that endoreplication promotes resistance against intrinsic and extrinsic stress (Lee et al., 2009) a feature that may be important to the HCs since their numbers are determined by proliferation rather than apoptosis.

Chromosomal variations in adult tissues are common, and aneuploidy (one form of heteroploidy) has been found in developing and adult cortical interneurons (Rehen et al., 2001; Kingsbury et al., 2005) as well as in chicken retinal ganglion cells (Morillo et al., 2010). However, in contrast to HCs, most aneuploid cells undergo caspase-mediated apoptosis (Voullaire et al., 2000; Rehen et al., 2001; Kingsbury et al., 2005; Rajendran et al., 2008; Zupanc et al., 2009; Peterson et al., 2012). Although the heteroploid HCs appear resistant to apoptosis, they respond to drug-induced DNA damage by phosphorylation of histone H2AX (γH2AX) and formation of γH2AX and RAD51 foci, suggesting a functional DNA damage response (Shirazi Fard et al., 2014a). Still the chicken HCs were able to enter S-phase and complete mitosis (Shirazi Fard et al., 2014b). This ability to enter mitosis in the presence of DNA damage indicates that these cells are able to withstand the effect of the DNA damage response (Shirazi Fard et al., 2014b, 2015). Furthermore, direct activation of p53 by its co-activator Zac1 neither induces cell cycle arrest nor apoptosis in HCs supporting the notion that the HCs are less sensitive to events that activate the p53 system, compared to other retinal cells (Shirazi Fard et al., 2015). These properties may associate with a cell that has the ability to become neoplastic, in fact conditionally inactivating the retinoblastoma *Rb1* gene in early murine retinal progenitors leads to degeneration of all retinal cells except HCs, which persist and divide with DNA damage resulting in poly- or aneuploidy (Donovan and Corbo, 2012).

## Horizontal cells and retinoblastoma

The childhood cancer Retinoblastoma is rare, with a reported incidence of 1 in 15–18,000 births. Since the discovery of *RB1* mutations, much effort has been invested into finding the cell-of-origin for retinoblastoma and understanding the carcinogenic mechanisms in those cells. Knock-down of *RB1* in human fetal retinal cells was sufficient to induce proliferation of cone PR cells and when grafted, these *Rb*1-depleted cone precursors were able to form tumors (Xu et al., 2014). This indicates that post-mitotic human cone precursors are sensitive to *RB1*-depletion and may represent cell-of-origin for retinoblastoma. However, a study performed on mice lacking Rb-family members, reported that HCs are able to re-enter the cell cycle, expand clonally and form metastatic tumors (Ajioka et al., 2007). Horizontal cells may therefore also be considered as a cell-of-origin for retinoblastoma. This poses the question why certain cell types are more prone to becoming malignant following loss-of-function of *RB1*. As discussed in this review, both the cone PRs and the HCs are among the first retinal cells to be generated during development and they are derived from the same multipotent progenitors (Suzuki et al., 2013; Boije et al., 2014). Whether a photoreceptor or a horizontal cell is the cell-of-origin for retinoblastoma may be less important from a mechanistic perspective. It is more important to understand the molecular pathways that distinguish the properties of these cells from other retinal cells, knowledge that may aid our understanding why these cells have a propensity for neoplastic transformation. In conclusion, HCs seem to have an atypical regulation or execution of their p53-p21 system during a limited period of the development that spans the time during which their neurogenic cell division occurs and that allows for genomic aberrations without triggering apoptosis. Such ability may be a factor that under challenging conditions, allows cells with *RB1* loss-of-function mutations to remain in the retina and to develop into cancer initiating cells.

## Summary

- There is a clear correlation between the morphologically defined HC subtypes and the classification based on molecular markers, which is evolutionarily conserved; the transcription factor Lhx1 is expressed in axon-bearing subtypes and Isl1 in axon-less subtypes.
- From an evolutionary perspective it seems that while most species have both axon-bearing and axon-less HCs, some animals may have lost the need for axon-less HCs due to adaptation to e.g., nocturnal lifestyles.
- FoxN4 and Ptf1a are crucial in establishing the competence to differentiate into a HC.
- While the Ptf1a-lineage is shared by HCs and ACs, the presence of Lhx1, which is expressed independently of Ptf1a, divides the lineage and thereby aids in the formation of HCs.
- The Lhx1-lineage gives rise to all HC subtypes and onset of Isl1 expression within this lineage drives the phenotypic change to axon-less subtypes.
- During development, committed HC progenitors undertake a bi-directional migration across the width of the retina during which non-apical mitoses occur in some species.
- The ability of HCs to undergo additional rounds of division in a differentiated state may be related to HCs being named the cell-of-origin for retinoblastoma.

## Author contributions

Conceived the review: HB, SS, PE, FH. Wrote the paper: HB, SS, PE, FH.

## Funding

Funding was provided by the Swedish Research Council, Medicine and Health.

### Conflict of interest statement

The authors declare that the research was conducted in the absence of any commercial or financial relationships that could be construed as a potential conflict of interest.
